# The pharmacist as safety net: an interview-based study of the intersecting dependencies between doctors and pharmacists

**DOI:** 10.1186/s40545-023-00536-1

**Published:** 2023-03-09

**Authors:** Courtney Addison, Denise Taylor

**Affiliations:** 1 Centre for Science in Society, Te Herenga Waka, Victoria University of Wellington, 22 Kelburn Parade, Wellington, 6012 New Zealand; 2 School of Nursing, Midwifery and Health Practice, CS 716, Wellington Hospital, Te Herenga Waka, Victoria University of Wellington, Riddiford St., Newtown, Wellington, 6021 New Zealand

**Keywords:** Doctor–pharmacist relationship, Prescribing, Dispensing, Roles, Aotearoa New Zealand, Qualitative

## Abstract

**Background:**

Social science research has demonstrated how health practitioners negotiate and contest professional roles and jurisdictions in practice, and in ways that reflect the power dynamics that permeate medicine. This article further explores these relational dynamics by examining how general practitioners (GPs) in Aotearoa New Zealand frame their working relationships with pharmacists.

**Methods:**

We conducted semi-structured interviews with 16 GPs from around the country. Interviews had a mean duration of 46 min, and were thematically analysed.

**Results:**

GPs saw and used pharmacists as a key source of information about both medicines and patients; thus it was not only pharmacists’ training and expertise, but also their community setting and patient proximity, that made them a useful resource to doctors. Furthermore, GPs framed pharmacists as a critical ‘safety net’ due to their role in catching errors and checking prescribing details. The pharmacy ‘safety net’ also came through in participants’ comments on discount pharmacies, which have introduced pronounced cost-cutting logics to Aotearoa New Zealand’s pharmaceutical landscape; in their reflections on these organisations, prescribers express the importance of robust pharmacy practice to their own work.

**Conclusions:**

Whilst the literature often foregrounds tensions in how health providers reinscribe their professional roles, this research highlights the interdependence that doctors identify with pharmacists, and their aspirations for working together. Both professional groups navigate a pressed health system that presents a set of common challenges to good medicines practice.

## Background

Primary care in Aotearoa New Zealand has long been framed as necessitating collaboration between doctors, nurses, pharmacists, and other healthcare providers. Appropriate working relationships between these parties is essential to meeting the many health challenges the country faces, including a suite of concerning pharmaceutical trends: rising antibiotic resistance [1, 2], increasing polypharmacy rates [3], and persistent inequities in treatable conditions [4, 5]. In light of such challenges, it is imperative to understand the social matrix of prescribing and dispensing. This article investigates one particular set of relations that shape Aotearoa New Zealand’s pharmaceutical landscape: those between doctors and pharmacists. Building on the social science literature on doctor/pharmacist role negotiations, we argue that these relationships are about more than defining and contesting professional roles and responsibilities—they are part of navigating a strained health system and supporting, as much as protecting, prescribers’ practice.

In line with international trends, medicines practice in Aotearoa New Zealand has seen a broadening role for pharmacists in recent years. In addition to traditional dispensing work, pharmacists might now undertake accreditation to perform medicines use reviews, administer emergency contraception and select oral contraceptives, and provide a range of immunisations [6] and other services [7]. As well as improving patient care and outcomes, this increasing scope is framed as potentially “reduc[ing] demands on other primary health care providers such as General Practitioners” [8]. General practitioners themselves report mixed, though increasingly favourable, responses to this proposition [9]. Many wish to work more closely with pharmacists [10], but have concerns about care fragmentation and potential confusion for patients [10, 11], as well as varying perspectives on which tasks and roles exactly are appropriate for pharmacists [9–11].

This shifting remit provides the backdrop for much of the research that investigates doctor-pharmacist relationships. These relationships are often marked by uneven power dynamics that position pharmacists’ expertise as secondary to doctors, even when it comes to medicines [12–14]. For example, writing in the Australian context, Broom and colleagues [15] note that although pharmacists hold extensive medicines expertise, “doctors maintain significant professional control over drug decisions, largely through the enactment of prescribing power”. Similarly, in New Zealand, the classification of prescription-only medicines positions doctors as pharmaceutical gatekeepers, and pharmacists primarily as providers of prescribed medicines. There is evidence to suggest that power differentials and a lack of mutual understanding between general practitioners and pharmacists inhibit effective collaboration [16]. Indeed, research in New Zealand demonstrates that some pharmacists experience a lack of respect for their expertise from the doctors they interact with [17], as well as a lack of awareness about what their role encompasses, and what value that adds for GPs [18]. Despite this, we know that in at least some contexts pharmacists already mediate important health information for patients [19–21] and serve as trusted community figures [22].^1^ In addition, other New Zealand-based research shows that clinical pharmacist facilitators integrated into primary care improve medicines use and care delivery [23].

Social scientists have often examined these and other interprofessional medical relationships in terms of professional ‘roles’ and ‘jurisdictions’. These accounts examine the hierarchies at work in healthcare settings, and how these are performed, naturalised, or negotiated so as to contest or conserve professional power, as well as associated forms of social and economic capital [24–27]. Doctors typically retain the major share of authority in these accounts, even when other medical professionals (e.g., nurses [24]) take on further training and responsibility. Clearly this bears on the expanding remit of pharmacists in Aotearoa and elsewhere. Professional hierarchies of course also materialise wider gendered and socioeconomic inequities [28, 29], and extend beyond the medical profession, enrolling cleaners, personal care assistants, and porters into a stratified labour organisation that often allocates the least value to the most patient-proximate work [29, 30].

Critically, these hierarchies not only structure social roles within medicine, but also infuse the epistemics of the field, privileging some forms of practice and expertise over others [28, 31]. For example, technical and diagnostic knowledge routinely takes precedence over care work (Ibid), while certain medical specialties carry more prestige than others [32]. These hierarchies also map liability and responsibility: in Mendoza and colleagues' [33] ethnography of addiction treatment in New York, pharmacists acknowledged their role as ‘the last line of defence’ in opioid dispensing, but ultimately saw prescribers as responsible for patients’ addiction and dependency.

Attending to this literature and our broader research project’s focus on how medicines are prescribed, dispensed, and communicated about, we ask how general practitioners (GPs) in Aotearoa represent their relationships with pharmacists. We show that general practitioners describe pharmacists as a source of patient and medicines information, and more crucially, as a safety net for their own prescribing practice. This usefully builds out existing literature on doctor-pharmacist role negotiations by showing how doctors value pharmacists in community practice settings, recognising their technical expertise and practical knowledge, and finding reassurance in the checks and queries they issue back to prescribers. In other words, general practitioners’ frame their own role as prescribers and care providers as mutually interdependent with pharmacists’ roles.

In what follows we outline our research methods then present our findings, delineating three key roles that doctors portray pharmacists as occupying: the pharmacist as complementary expert; as patient-proximate; and as safety net. We also analyse a strand of commentary that arose in our interviews concerning the arrival and impact of large, corporate, discount pharmacies in Aotearoa, which became a locus for doctors to reflect on the role of pharmacy in relation to their own practice.

## Methods

This paper draws on interview-based research conducted from May to June 2022, as part of a broader project investigating how information about medicines moves between patients, pharmacists, and general practitioners. To explore how GP’s approached prescribing decisions and understood their relationships with patients and pharmacists around medicines, we elected to use an interview methodology. Interview methodologies are well-suited to research that investigates people’s thoughts and experiences, and the semi-structured format accommodates emergent findings that might not be anticipated in the original research design. The project received ethics approval from the Victoria University of Wellington Human Ethics Committee (#28324).

Given our aim to solicit general practitioners’ views specifically, we followed a purposive sampling strategy and recruited from a national pool, so as to gain cross-sectional insights from different geographic, socioeconomic, and institutional settings. Using the Medidata database of general practitioners, we issued an invitation for interview participants that reached 1,331 recipients. Of these, 25 people registered their interest via the supplied link, and 16 followed through to an interview. Our only selection criterion was that participants must be currently practicing general practitioners, as all 25 initial respondents were. Recruitment stopped when participants stopped opting in to the study.

While most of our resulting sample worked full-time in general practice, some of our interviewees worked part-time, as locums, or combined their general practice work with, e.g., working for hospice. Of our sixteen participants, 10 were female and six male. Eight were based in one of New Zealand’s three major cities, six in a smaller city or large town, and two rurally. Thus, while our sample is not representative, it does encompass a range of professional and regional experiences and clear themes were evident across the data set.

Interview guides were developed from the overall project’s aims, and included questions about participants’ professional backgrounds and contexts, prescribing practices and views on medicines, relationships to pharmacists, and perspectives on the New Zealand health system. In keeping with the semi-structured interview process, we allowed these guides to steer our conversations without dictating or unnecessarily limiting their course. The interview procedure was not adjusted in light of emergent findings, so as to ensure maximum comparability across the data set. However, the interview guide was adapted in some cases to suit individual participants’ time availability.

The authors conducted all interviews via Zoom, between May 1 and June 30 2022. Interviews lasted from 24 to 68 min, with a mean duration of 46 min 30 s. Typically, shorter interviews were those conducted during participants’ lunch breaks, and longer ones were conducted on participants’ days off or in the evenings. All interviews were audio recorded using the computer’s inbuilt recording software, then professionally transcribed verbatim. The original audio files and resulting transcripts were allocated pseudonymised alphanumeric file names (linked to identifying information in one securely stored spreadsheet) and stored in the University’s secure cloud storage system.

Although Denise Taylor training as a pharmacist could potentially be expected to influence how interviewees spoke about the pharmacists they interacted with, we did not note any discernible differences in the results collected by each interviewer, and neither had any existing relationship with any participants. It is possible that conducting interviews via Zoom also mitigated the extent to which the researchers’ positionality shaped interviews. We were known to participants’ primarily by our qualifications, professional roles, and association with a respected funder, with few cues as to our respective physical presentations beyond the shoulders-up Zoom window.

Our data analysis was conducted by (Courtney Addison) June through October 2023, and checked and discussed with Denise Taylor. Our analytic process was grounded in the constructivist tradition of Corbin and Strauss [34], which acknowledges the role of the analyst in meaning-making, and followed a Reflexive Thematic Analysis process [35]. This began with familiarisation with the data set (all interview transcripts), followed by iterative, open coding of the corpus. Codes were then reviewed and grouped into themes, which were themselves reviewed against the transcripts. The review process enabled refinements to the theme, so that, for example, the theme ‘Doctor/pharmacist interactions’ became ‘Sharing information about patients’, ‘Seeking medicines information’, ‘Correcting mistakes’. To check the validity of findings, the authors discussed transcripts and codes from their distinct disciplinary perspectives (Anthropology and Pharmacy, respectively) and also compared findings against the data set from the other arm of this project, which consists of interviews and observations from a community pharmacy. This confirmed, for example, that doctors were using pharmacists in the ways they described in the interviews reported on here, and that pharmacists were indeed picking up mistakes as interviewees report.

## Results and discussion

### Pharmacists as “complementary experts”

A key finding of this research was that doctors value pharmacists’ and their knowledge in multiple ways: for their expertise, proximity to patients, and for their role as a safeguard to prescribing. In the following three sections we explore these findings, and argue that collectively, they demonstrate how pharmacists act as a ‘safety net’ for doctors.

In the first half of our interviews, we guided doctors through questions about their prescribing practices, before moving on to ask about their relationships with their local (or other) pharmacist/s. While few interviewees spoke about pharmacists during those earlier exchanges, once asked, almost all subsequently affirmed the importance of their relationships with one or more local pharmacists. They reported communicating frequently with pharmacists, for a range of reasons. One commonly reported reason was to seek out information about specific medicines. One participant, when asked how much interaction she had with her local pharmacy team, explained:

A lot, because we’ve got an adjoining pharmacy to our practice, so I ring them most weeks. I’m often in there, either asking questions or dropping off a prescription or getting something explained… when I had some elderly patients in rest homes and they couldn’t swallow that well [I asked] which of their medications could they crush and then put in a dairy product, so whether they interacted with calcium. (205AK)

Continuing these reflections later in our interview, this participant added, “it’s great to have those collegial sorts of complementary experts”. This one brief comment speaks simultaneously to the affect of these encounters (collegial), their functional role (complementing GP’s practice), and a recognition of pharmacists’ insight as expertise. The interviewee’s nod to the ‘adjoining’ pharmacy supports suggestions that physical proximity between doctors and pharmacists can support mutual understanding [16, 23, 36].

Another doctor explained, “I will phone them for advice when I’m really trying to read a prescribing guide and I just can’t get my head around it—something I’m not familiar with—I will ring and ask” (35CH). Another doctor, who was in her third year of practice, spoke about some patients’ preference for branded medicines as opposed to the government funded generics, telling us, “I definitely rely on the pharmacist to help me navigate that, if the patient couldn’t or wouldn’t take the generic—what other ones are available”. A senior GP made a similar remark, stating:

…if there’s a medicine and I’m not sure whether there’s any alternatives, I might give the pharmacist a quick call, just to update on whether it’s still available, or whether there are other things you can use instead. So, I kind of use them as a resource, as well. (00PR)

In comments like these, general practitioners recognise and defer to pharmacists’ distinct knowledge of the medicines that are prescribed in the clinic. This offers a potential contrast to accounts of how doctors source information about new medicines, in which pharmacists go unmentioned [37, 38]. However, it is also consistent with some other local findings that doctors value pharmacists’ medicines expertise [11].

There is a logistical aspect to this relationship as well, as stock issues comprise a significant portion of doctors’ and pharmacists’ interactions. This often entails discussion about what to do when certain medicines are temporarily unavailable.

They leave a wee note, ‘oh, this is out of stock’. That’s a common one, ‘this one’s out of stock, suggest this alternative’—which is useful, because that was driving me nuts a little bit, for a while. There were so many things out of stock, and it would come back in stock, and then it would go again—blood pressure pills and contraceptives and things… HRT (35CH)

Here, it is not only that the pharmacists are keeping their local medical teams up to date about stock supplies, but also that they are able to suggest alternatives to make the work of prescribing easier. Another GP noted that she would consult with the pharmacist about what her prescribing options were:

I would call to check [...] some things that I had heard were not in stock, or say a while ago this non-antibiotic ear-drop that can help people that get a little chronic irritation went out of stock, so I called a pharmacist to talk about what they’d been advising people to do over the counter. (134PR)

A rural GP explained how their nearest pharmacist would regularly visit to replenish the store of essential medicines they kept on hand in the practice. Like other interviewees, he pointed out that stock shortages had been particularly acute during the pandemic:

We’ve had difficulties overseas getting medicines here, so they’ll give us a bit of an update on when medicines have come back in stock, or whatever. Often, if we prescribe a medicine that we can’t actually access, then they’ll contact us, so that we’re getting a kind of alternative medicine. (00CA)

For these doctors, pharmacists possess both specialist medicines knowledge and a pragmatic command of the local medicines landscape, such that if a particular product goes out of stock, they are able to both alert prescribers, and suggest safe alternatives.

Importantly, pharmacists’ expertise was not only “a resource” for the doctors themselves—many also sought to encourage patients to make use of their pharmacists’ extensive knowledge. One doctor explained that she tried to direct patients to the pharmacy for certain health concerns:

The other thing is just trying to get patients to go to see their pharmacists for minor things, a lot of people don’t realise that they can do that, so it’s often just suggesting for the nurse to say to the patient, ‘well why don’t you go to your pharmacist, talk to them about your blocked nose’. (29NP)

Another GP suggested that pharmacists were the best people for patients to discuss side effects with. “Do we [doctors] broadly talk about side effects?,” he reflected, “Yeah. Yeah, we do—to some extent. I think we try and do it in a broad-brush way… Do we do it well? I don’t think we can do it as well as a pharmacist” (25JD). GPs who worked or had worked with specialist clinical pharmacists specifically were also advocates for more hands-on involvement between those practitioners and their patients. Many recalled how useful it was to have medicines reviews performed for patients, and one suggested that the clinical pharmacist at their past practice had represented an independent source of medicines information to patients, where cost was seen as a potentially compromising factor as well as a barrier to access.

### Pharmacists as patient-proximate

During our interviews we asked doctors whether they felt that pharmacists saw a different side of their patients to what they saw in their practice. Most felt they did. One remarked:

They might actually even see the patient more than I do. They might be able to answer more questions, because a patient might walk out [of the doctor’s office] and then have a whole bunch of questions, and the pharmacist could go through that with them. Every health professional sees a different side of the patients; we all have our own lens to see the patient with the training that we have. So, yeah I think they have definitely a different, really helpful insight. (105TA)

Another interviewee similarly noted that in the process of getting from the doctor’s office to the pharmacy “the information had time to sink [in], and sometimes they [the patient] come with different questions, because they had 20 min until they get to the chemist, or a day, and they can come and ask, ‘well, I wasn’t sure about ABC’” (45AB). Another pointed out that pharmacists might meet patients’ whānau (family) and gain additional insight from those interactions (245CH). These comments speak to both the pragmatics of accessing different health services, and the more nuanced observation that different health providers will inevitably see and reach patients through the rubric of their own training and positionality. In addition, two people highlighted the different dynamics in the pharmacy, which they saw as more public and transactional, and, therefore, *less* conducive to sensitive conversations.

These different vantage points mean that pharmacists can also offer insights about patients that GPs might not be privy to. Our participants told us that they would sometimes collaborate with pharmacists on their shared patients, particularly those they suspected might be misusing or struggling with their medicines. As one doctor explained, while talking more broadly about their interactions, “the other reason we might communicate is drug-seeking, for people who are picking up, and they might try early pick-ups and just occasional patients to keep in touch over” (35CH). Similarly, a participant recalled, “I’ll ask them, ‘hey, this person is coming for their Tramadol?’, or whatever, ‘how many pick-ups have they been doing?’ Stuff like that.” (105TA). In these contexts, GP’s combine their understanding of what patients *should* be taking with pharmacists’ hands-on knowledge of what medicines they are actually collecting, with a view to preventing potential misuse. Another doctor offered a different insight into the challenge of addiction, speaking about how they sought to prevent medicines misuse for people with known addiction problems by prescribing appropriately:

Sometimes I will have a chat with them [the pharmacist] if I’m just trying to find out a particular patient—so, for example… where we’re doing close prescribing of some kind, so someone with an addiction problem, and we’re trying to sort out—making sure that they’re given the correct amount, and not given too much. (00PR)

While general practitioners know and have records of what they prescribe to which patients, they rely on pharmacists’ insight into whether and when those patients are collecting their medicines. By exchanging this information, they develop a fuller picture of patients’ medicine use.

GPs also reported communicating with pharmacists about those patients who might be struggling with their medicines or their health more broadly. These patients were often elderly. Sometimes, these exchanges were as simple as a piece of advice relayed from the pharmacist to doctor: “they might just say, ‘I actually think blister-packs would be useful for this patient—can you change the prescribing?’” (35CH). Another offered a similar perspective, saying, “we do communicate on patients; the difficult patients—the patients that always lose their prescriptions, or always get muddled with their medication—the ones that need to be blister-packed, et cetera” (205AK). One participant spoke about how their own concerns might prompt them to reach out to the local pharmacist, and then reflected on how the pharmacists’ own vantage might grant them insight into patients’ challenges:

GP: It’s not uncommon, if I’m having concerns about elderly patients, and not sure whether they’re taking their medicines properly, I might have a conversation [with the pharmacist].

INT: So, do you think the pharmacy team see a different side of the patient to you?

GP: I suppose they do. Probably just from the point of view of the medications. They’d be more in-tune with whether the patients are complying with their medicines, for example. So, they would pick up if a patient seemed very confused and kept on either missing prescriptions, or coming in. So, one of the things that came up as an issue, particularly with my patient population, is when they’re starting to get cognitively impaired, so they may be able to identify that quicker than I would. (00PR)

In these exchanges, patients’ medication needs are not only what must be prescribed to address their health problems, but also what supports need to be put in place to enable patients to differentiate between their medicines and remember which to take when. The doctors’ appointment is not the only site where this information is gleaned: the process of handing over their medicine in the pharmacy, or seeing an uncollected package on the dispensary shelf also offer insights into patients’ behaviour and well-being.

One of our participants, a senior rural GP, also elaborated on how this collaboration could play out in more informal but arguably more powerful ways. “There’s a wider role to that” relationship, he told us, recalling a recent incident in which the pharmacist located next to his practice reached out to ask about a patient they were concerned for. He recounted their conversation:

[Dr], I haven’t seen Mary Blogs—she hasn’t been in, she hasn’t picked up her medication—have you seen her? No, I haven’t. Talk to the nurses, rang—don’t get a reply. Okay, put her on my visit list, I want to swing past later in the day. I’m talking about something that happened three weeks ago. There she was on the floor with a broken hip—probably been there for a couple of days, because she was super-cold. She looked at me and said, “Doctor, I knew you’d come.” (185KU)

In his recounting of this story, the doctor attributed these relationships to his context operating in a rural practice. His familiarity with the pharmacist next door, and their mutual familiarity with the elderly patient in question, were borne of lengthy histories working in same community—albeit a community that is now more city-fringe than rural, and with a rapidly changing demographic. Another doctor shared a story that similarly highlighted how familiarity can translate into important forms of care:

One day, it was a Saturday, I got called about a blood test result for one of my patients… I wasn’t at work, and it was really awful, like this INR number was really high, which meant that if that person had fallen over they could have potentially had a massive haemorrhage somewhere, like a brain bleed or an abdominal bleed or something, it was the level you have to go to hospital for, but it was a Saturday, I was in town and I wasn’t going to be able to make it home, but I called our pharmacy… they were open on a Saturday and I said ‘can you get in touch with this patient or can you give me their number?’, and because they knew me and they knew the patient, they had the telephone number for the patient and so I could then call the patient and say ‘hey you need to go to hospital’… and it was fantastic and I suppose that’s more that personal, they knew that patient, they knew the details of the patient, they knew me so they knew that I wasn’t some rando off the street saying ‘I want this patient’s details’, so it was that nice little closed loop (276WL)

These accounts attest to the value—both essential and unmeasurable—of long-term relationships between medical providers and their patients, and of close working relationships between doctors and pharmacists, who see the same patients, albeit in different settings. Getting a patient the medicine they need requires both pragmatic and specialist knowledge about the patient, their diagnosis, and the medicine/s being prescribed. They also illustrate the fundamentally relational nature of pharmaceuticals, highlighting how prescribing practice is shaped not just by doctors, or even doctors and pharmacists, but by the interaction between them, over time and in relation to different patients and settings.

### Pharmacists as a “safety net” for prescribers

Perhaps the most fundamental role pharmacists play for GPs is that of the ‘safety net’. Here, we borrow a phrase used by several interviewees, which captures how pharmacists’ guard against potential prescribing errors. Dose changes, medicines interactions, and changes to patients’ prescribed medicines were all subject to pharmacists’ expert eye and patient knowledge.

Pharmacists are really good at calling us with prescription anomalies, like if a medication is dropped off, and it’s not clear that it’s been stopped, they’ll often call and check if that was on purpose, and they will sometimes call with medication interactions that we haven’t noticed, which we appreciate. I’ve had one episode where that was really helpful where someone’s as-needed medicine wasn’t—they were using it as-needed, but it was prescribed the year before, so it was really far down the list, and I didn’t see it, and then I prescribed them an anti-fungal for their really bad fungal infection on their body that creams weren’t going to help, and then I stopped their cholesterol medicine which they were on regularly while they were on it, and then I sent it off, and the patient… he’d re-started the medicines from 2021, but then the pharmacist asked him if he’d been taken any extra medicines than the ones on his list, and then he mentioned this, and there’s a big interaction between those two medications. So, I got a phone call about that. So, it’s nice having that extra thing, because you’d forgotten to double-check over-the-counter medicines at the time. It’s nice having that back-up of someone else asking when they’re dispensing it. (134PR)

This excerpt captures a common phenomenon—complex prescribing decisions for patients on multiple medicines—and highlights how easily-made oversights are corrected for when a script makes its way into the hands of an attentive pharmacist. All but one of our participants described interacting with pharmacists in similar ways. For example, another doctor reflected:

Both our pharmacies are right next to the practices, and there’s another one in town, so occasionally you’ll get a note—‘did you mean to prescribe this dose?’ I’ll say, ‘yes, I did’. They’re just checking. They might say, ‘the patient is already on this—just so you’re aware—had you thought about these interactions and things?’ (35CH)

The aside here (“they’re just checking”) hints at the power dynamics that contour doctor-pharmacist relationships, and tensions over professional boundary crossing that other work has documented [15]. While some research shows that pharmacists are stereotyped into a ‘policing’ role (Ibid), here a senior doctor represents the scale of intervention as both more modest and constructive. Furthermore, in Aotearoa, general practice and community pharmacy settings account for a significant proportion of medication errors [39]. A mutually attentive relationship between GPs and pharmacists may be an important safeguard in such a context.

We asked another senior doctor, whose general practice employed a pharmacist and who worked closely with another in hospice contexts, if this proximity meant prescribing decisions were more “collaborative”. He replied fondly, “They’re more a bargaining thing really. She [the pharmacist] wants to cut it down and we want to release the dose… they’re a delightfully conservative group of people” (25JD). Later in our interview we asked if he used the literature to keep abreast of emerging data on side effects, and he replied, “Yeah, but nothing like your pharmacist there who can just lean on us all the time and doesn’t let us make a move out of place.”

In some cases, the close attention doctors received from pharmacists was the exception rather than the rule. Another doctor described her appreciation for one particularly engaged local pharmacist:

[He] was the only one really routinely who would call me and be like, ‘oh [Dr] did you mean to do this’, like [laugh] he’s *very* conscientious, which is good, I think, that’s what I imagine pharmacists are meant to do, check that actually did we mean to do what we said we did… So [that pharmacy and I] had quite a good relationship, so they could talk to me and say ‘did you mean to do this or did you not?’ or, ‘that dose is a bit funny, it’s different to the one they got at the hospital, is that what you meant to do?’, and that was really—I wanted that, I want that from all the pharmacies but I didn’t get that with—most other pharmacies didn’t really communicate, it felt like a one way street, I would send a prescription into the ether and hope that what I wanted to have happened has happened. (27WL)

This same interviewee concluded her thoughts with reference to safety:

As I say… [they] were totally happy to tell me or ask me, check what I’d prescribed, ‘oh is that what the hospital’, or ‘did you mean to do that’, you know and that’s to keep the patients safe, at the end of the day that’s why they do it, not because they want to hassle me, it’s to keep the patient safe. Which is what I want too. (134PR)

In a powerful statement of common purpose, this doctor frames the regular checks and queries she receives from her local pharmacist both humorously (she is clearly somewhat entertained by his level of scrutiny) and as genuinely high stakes—as a matter of patient safety.

A rural doctor explicitly articulated what many of our participants alluded to as he walked us through the occasions on which his local pharmacist would reach out to him.

The pharmacist will always be checking the medicine… they will say, ‘have you stopped this particular pill, because normally this person is on this particular pill?’, or ‘has that changed?’, or a dose that we have made, and forgotten to notify them about. They will phone and check that. So, that’s a great thing, and of course, they will also—if they believe that something may be unsafe, or the person may have an allergy or something—it doesn’t happen very often, but they’ll contact us with that, and *I see that as a great safety net for patients, and for me*, because we can all make mistakes, and it’s always good to have someone watching your back (00CA, emphasis added)

In expressing the pharmacist “safety net” as in service of both patients and doctor, this participant illuminates how central pharmacists’ work is to the smooth running of general practice: pharmacists not only serve patients, but also provide indispensable checks and assurances to prescribers. In this formulation, good prescribing practice and medicines use is an emergent property of open, attentive relationships between prescribers and pharmacists.

This interpretation of pharmacist as safety net is less a reflection of the group’s professional remit, and more of the health system context. Although questions of responsibility and liability inhere in both doctors’ and pharmacists’ roles,^2^ we read the ‘safety net’ motif as a form of commentary upon health system pressures, and prescribers’ resulting vulnerability. For example, our participants routinely spoke about appointment time limits as the major barrier to best practice prescribing—they lamented how hard it was to hear a patients’ complaints, reach a diagnosis, and render a treatment decision in the 15-min window that is the default in most practices. This was compounded by issues of cost and access: some doctors cited patients who struggled to afford the cost of a doctor’s appointment, and so would ‘save up’ their concerns to address multiple issues in one visit, further exacerbating time pressures within the consult. Against this backdrop, the pharmacist safety net not only protects individual prescribing decisions, but also a broader health system under strain. This theme found expression elsewhere in our interviews, when doctors spoke about the emergence of bargain pharmacies.

### The impact of budget pharmacies

Discount or bargain pharmacies (“fast food pharmacy”, to one interviewee) have been operating in New Zealand for several years, with supermarket chain Countdown opening in-store pharmacies in 2012, and Australian company Chemist Warehouse entering in 2017 [41]. These companies follow a loss-leading model, wherein they absorb the $5 dispensing fee collected by government, seeking instead to make up that revenue through the retail branch of their outlets. Though these businesses promote themselves as serving patients’ interests by making prescription medicines free, many of our participants were apprehensive about their effects on patient health and the medical sector.

Often, interviewees’ anxieties centered on the high-volume business model of discount pharmacies, which doctors worried would compromise the more patient-centred aspects of pharmacy. For example, the following exchange took place between Denise Taylor and a GP:

Dr: Unfortunately, my opinion is sometimes you get what you pay for.

Int: Could you expand on that, please? I think I know what you mean, but –

Dr: Look; sometimes they [patients] need [the] explanation of the chemist, and when the chemist—again, in my opinion—I don’t know how much they’re being paid, but they are so over-loaded, and I’ve been there [the discount pharmacy]—they are so over—they don’t have the time to discuss with the patient, the medication. They don’t have time to explain how to take it, because we don’t write everything—there’s not enough place on the script to write all the information… at first… everyone wanted to go and get the free one, but now we see a shift, because they want to get the chemist to actually have a talk with them. Sometimes, people can accidentally get the wrong medication. Don’t get me wrong, we’re all human, we make mistakes sometimes. Having the chemist as another area that can stop a mistake from happening is very important.

Int: So, a pharmacist that knows the person’s history –

Dr: Yes, knows the patient. It’s like Cheers, you go to the pub and everybody knows your name. It is very important… I believe it is important to have a chemist who knows you and gives you the time you need. (45AB)

This doctor’s personal observations from their nearby discount pharmacy enfold their concerns about whether patients’ needs are being met, *and* whether the ‘safety net’ that prevents medical providers’ mistakes is functioning appropriately. Indeed, in this account, rather than pharmacists catching errors, they are potentially making them. Here, a business model predicated upon high volumes of scripts is explicitly linked to an inability to provide optimal care for patients and support for doctors. Some speculated that discount pharmacists might not form the types of relationships that facilitate good medical care:

There’s definitely an advantage to having pharmacists who are actually in the community, known the patients for years, get them the appropriate advice, because as a doctor, sometimes we prescribe and we don’t necessarily go into detail about how to take the medication, or whether to take it before or after food; that sort of advice—very helpful from a pharmacist directly. I’m not sure if you get that with the big chains, because those guys just push the medicines out. So, you kind of lose that personal touch, as well as they’re not local (155CH)

Like the comment above, this reflection highlights the interdependence of pharmacy and general practice, indicating that the impacts of pharmacy practice are felt well beyond the pharmacy doors. It also demonstrates how existing pressures on general practice, particularly the limited time available for appointments, shape doctors’ perspectives towards this new model of pharmacy, which they see as ill-equipped to give patients the time that doctors themselves cannot.

Discount pharmacies were also seen as *indirectly* compromising patient care by possibly driving smaller, established pharmacies out of business. A doctor from one major urban centre remarked:

I’d like to see more of a level playing field, but I don’t like the way that the bargain chemists are sucking the oxygen out of these community pharmacies, like ours, that does a fantastic job *supporting us* in general practice, and *in doing all of those things individually for the patients* like blister-packing and all the little difficult things about—all the little interactions of medications—they’ll do all that, whereas the bargain pharmacies or the zoom pharmacy… the Chemist Warehouse, et cetera, where patients want to shop around to get a free prescription—they don’t provide that same service (205AK)

Again, this respondent links both care for patients with support of doctors, highlighting the interdependencies that allow different medical practitioners to operate effectively. Another GP observed that the arrival of these businesses,

is probably good for the patient, but it’s not good for us, because of all the reasons we’ve talked about, with the relationship with a local pharmacist. If we send the medicines off to the bargain chemists, we don’t know whether they’ve picked them up. We get no feedback. (00CA)

This comment introduces a perspective shared by several of our participants: that free prescriptions actually *are* a valuable offering for patients who often struggled to afford their medications. One doctor thought through this as they spoke:

I mean, for us as GPs, I guess we don’t look at the financial issues for the pharmacy owners. I’m imaging that the Chemist Warehouse and Countdown pharmacies and that sort of thing are taking away obviously a lot of the clientele from the older models of urban pharmacies. So, they’re a financial threat, but that’s not something—I don’t know, actually, I am a little bit torn about it; it’s good for the patients to not have to pay, so you can suggest they go to Chemist Warehouse and Countdown pharmacies, but on the other hand, that could mean the end of suburban pharmacies. So, I’m a bit torn about trying to support local, and people who provide an excellent service, but at the expense of—the patient can go somewhere and get a cheaper product, so it’s hard to know. (25AK)

The ‘local’ emerges in these remarks as a critical substrate of familiarity and solidarity. This echoes Duckett's (2013) findings from community pharmacy research in London, where pharmacists who had lived and worked in their pharmacy area felt better able to build rapport with patients, even across other forms of difference, such as ethnicity. Another respondent recalled how starkly patients’ financial circumstances constrained their ability to access care:

…it makes sense for so many people here who can’t afford their prescriptions; if you’re on eight medications and it’s $5 per item, you’re very much penalised for being unwell. Before we had those prescription-free pharmacies, we’d sometimes have people walking in asking the receptionist which medications do you think they should get [sic], because they’d have a certain amount of money to spend on it, so they’re not going to buy them all. Then, she’d be talking to the nurses about what they can do, and you’re trying to triage them a little bit. So, it’s been a really important change for some of the patients here, to be able to actually pick up their whole prescription. (134PR)

Attuned to their patients’ limited financial means, these doctors recognise that prescription costs, even when subsidised, represent a very real barrier to healthcare access. The equity implications of this dynamic were echoed by another doctor:

It’s just ridiculous how cheap they are, but I don’t recommend them, because I far prefer, like I was saying, the—you know, the owner‑pharmacy because they’re just so much more caring and—I don’t want to say knowledgeable, but more—I don’t know. They just seem to be more caring and put more into it. And people know that as well. Like, people will stick with their chemists... unless they’re low income, you know, or a young person or something and they’re going to get the—not have to pay the script fees, then you know, fair enough if they’re going to go to Bargain Chemist (296NP)

This comment articulates a potential predicament for medical practice and healthcare more broadly in Aotearoa. Doctors fear that discount pharmacies provide substandard care, but they also recognise that their low-income patients may be unable to go elsewhere. They also expose a dilemma: if discount pharmacies improve patient access, but that access is to medicines that are stripped of necessary expert mediation, then is this really in patients’ best interests? Interviewees were often deliberate about framing their concerns as ‘suspicions’ or ‘opinion’ rather than evidence-based knowledge, and indeed, there is a clear need for future research on this issue. For now, we suggest that GP’s anxieties bespeak a real concern with the vulnerability of general practice to developments in the world of pharmacy. Doctors are acutely aware of the constraints on their own practice, particularly the cost of appointments and the limited time these are allocated. Seen from the day-to-day of working amidst such constraints, pharmacy represents an indispensable safety net—but one that could be undone by corporate incursions and a lack of policy support.

Importantly, some of our respondants reported positive experiences with their nearby discount pharmacies. One doctor (25AK) pointed out that their local budget pharmacy was staffed by locals, and that sometimes smaller, independent pharmacies also experienced a high turnover of staff. The doctor who had described patients asking the practice receptionist which medicines they should prioritise, reported:

I have been pleasantly surprised by some of the local ones—the bigger—is it Bargain Chemist, and Chemist Warehouse and stuff; we’ve still gotten phone calls from their pharmacists, with queries and insights for their prescriptions. So, it’s definitely reassured me that it’s less of a factory process, and that there are still pharmacists there who can build relationships with patients. (134PR)

These experiences highlight the complexities of the pharmacy landscape: small pharmacies are not immune to staff turnover and the need to break even as a business, and cost cutting pharmacies are not necessarily unable to deliver good quality patient care. Certainly, their comments speak to the need for further research in this space.

These concerns also have historical precedent. Pharmacy in Aotearoa has always encompassed both medical service provision and retail sales, tightly binding up social and economic interests. As social pharmacist Pauline Norris (1995) writes, when British pharmacy company Boots began making inroads in New Zealand in the 1930s, local pharmacists argued that they were foremost service providers (as opposed to businesses), and should therefore be exempt from the market pressures that international competitors would introduce. These concerns are reprised in contemporary anxieties—here, doctors’—over the potential impacts of discount pharmacies on not only local pharmacy, but healthcare more widely.

## Conclusion

This research highlights the intersecting dependencies between doctors, pharmacists, and patients in Aotearoa New Zealand. While tensions and power imbalances between prescribers and pharmacists are well-documented [10–13, 15, 24, 25], our work adds another perspective by foregrounding doctors’ accounts of working with pharmacists. They see the latter group’s medicines expertise as a benefit to both patients and prescribers, and their close-up patient knowledge as a further asset to their medical practice. More fundamentally, we have shown that pharmacy represents an important safety net for doctors who feel systemically constrained in their efforts to care for patients. These findings agree with earlier work that highlights pharmacists’ efficacy in picking up medication errors, making dose adjustments, and monitoring patients’ responses to medicines in other settings [43]. This lends weight to calls for greater recognition of, and remuneration for, this necessary labour that pharmacists perform [44].

Our findings also offer a useful complement to the literature on roles and jurisdictions, by highlighting the collaborative work and collegial relationships that support good medicines use. This literature documents an expanding role for community pharmacists over the past 20 years, and shows that pharmacists have been more supportive of this expansion than doctors, [9], albeit with differences regarding the specific activity types in question [11]. In a context where some pharmacists feel that doctors do not value their roles or expertise [18], our findings offer a modest but important contribution, showing how general practitioners themselves articulate the importance of their pharmacists’ expertise to their practice. This also has policy implications, suggesting that the full complement of pharmacists’ labour ought to be recognised in funding models (c.f. [45]). Taken alongside our own research-in-progress with pharmacists, it also suggests devoting policy attention to enablers of collaboration between doctors and pharmacists, including opportunities for co-location, and systems that enable correspondence and information sharing across practices and pharmacies.

Building on research into how roles are defined and professional boundaries negotiated, we suggest that in articulating pharmacists’ roles, doctors also outline the limits of their own. In medicines use, the pharmacists and doctors’ roles are inherently relational, produced through their interaction around prescribing decisions and their aftermath, rather than preceding that interaction. Furthermore, doctors identify common ground with pharmacists through their attention to time constraints and the rise of discount pharmacies. Thus, rather than *only* being about roles and professional competition, we read these accounts as commentary upon much wider, structural issues with New Zealand’s health system and society more broadly.

To be clear, we don’t suggest that doctor-pharmacist relationships are free of tension. Our sample is self-selecting, and undoubtedly skews towards those doctors who have a strong enough interest in pharmacy and prescribing to dedicate an hour of their time to this research. These limitations mean this work should be taken as a prompt for further research, rather than as a source of generalisable insights itself. In addition, interview-based research is useful for learning how people think about specific issues, but this thinking does not always correspond to practice. Further research should, therefore, investigate pharmacists’ experiences of working with doctors, and explore how these relationships function in practice. For example, pharmacists also face significant time pressures in their work [8], and there is some evidence that pharmacists may be less satisfied with their relationships with GPs than vice versa [10].

However, in a context where the health workforce is under strain and medicines comprise an important part of medical care, it is also essential to understand what characterises good doctor-pharmacist relationships, and how these parties aspire to work together. The pharmacist safety net represents a source of protection for doctors, but it is also a symptom of a health system that leaves doctors, patients and pharmacists vulnerable to each others’ limitations, and those of their broader social setting.

## Data Availability

Our data (interview transcripts) are not publicly available, and our ethics approval does not contain provisions to make them so.

## Footnotes

1 Our own preliminary unpublished research suggests that both of these findings hold true in at least some New Zealand pharmacy contexts.

2 In 2016 the Pharmaceutical Society of New Zealand provided feedback to the Ministry of Health on prescribing and dispensing regulations. They wrote that “poor prescribing, in the sense of writing a prescription and meeting the legislative requirements of that prescription, is extremely common. Unfortunately it falls by default to the dispensing pharmacist to pick up these errors and omissions and ensure they are corrected. We would like consideration given to how legislative documents describe the responsibility for prescribers to meet the legal requirements of ‘the prescription’” (40).
